# FMRP-mediated spatial regulation of physiologic NMD targets in neuronal cells

**DOI:** 10.1186/s13059-023-03146-x

**Published:** 2024-01-23

**Authors:** Tatsuaki Kurosaki, Xavier Rambout, Lynne E. Maquat

**Affiliations:** 1 Department of Biochemistry and Biophysics, School of Medicine and Dentistry, University of Rochester, Rochester, NY 14642 USA; 2 Center for RNA Biology, University of Rochester, Rochester, NY 14642 USA

## Abstract

In non-polarized cells, nonsense-mediated mRNA decay (NMD) generally begins during the translation of newly synthesized mRNAs after the mRNAs are exported to the cytoplasm. Binding of the FMRP translational repressor to UPF1 on NMD targets mainly inhibits NMD. However, in polarized cells like neurons, FMRP additionally localizes mRNAs to cellular projections. Here, we review the literature and evaluate available transcriptomic data to conclude that, in neurons, the translation of physiologic NMD targets bound by FMRP is partially inhibited until the mRNAs localize to projections. There, FMRP displacement in response to signaling induces a burst in protein synthesis followed by rapid mRNA decay.

**Supplementary Information:**

The online version contains supplementary material available at 10.1186/s13059-023-03146-x.

## Introduction

Studies that have connected the mechanisms of nonsense-mediated mRNA decay (NMD) and the translational repressor FMRP in mammals have almost exclusively utilized non-polarized cells. Notably, NMD, like FMRP expression, typifies all mammalian cells [1, 2]. It was recently found that FMRP is, as a rule, enriched on most if not all NMD targets via binding to the essential NMD factor UPF1 [3], if not also by direct FMRP binding to GC-rich and structured sequences [4]. Additionally, in SH-SY5Y cells, minimally one-third of FMRP-bound mRNAs are NMD targets [3]. This inextricable connection between NMD and FMRP signifies that the efficiency of NMD is dampened from what it would be in the absence of FMRP, regardless of cell type. This conclusion is consistent with data demonstrating that NMD is rarely, if ever, 100% efficient [5, 6]. In addition to NMD downregulating the already low levels of an estimated one-third of mRNAs that derive from mistakes made during transcription or pre-mRNA processing, NMD downregulates the expression of ~ 5 − 10% of physiologically important mRNAs during adaptation to changes in the cell environment, e.g., during differentiation and development [6–8]. Physiologic NMD targets, which are the focus of this review, can harbor an upstream open translational reading frame (uORF) or result from regulated, i.e., constructive, alternative pre-mRNA splicing and/or 3′-end formation [1, 8, 9].

Given that inherited deficiencies in either an NMD factor or FMRP can result in intellectual disabilities [7, 8, 10], it follows that NMD and FMRP should be studied in cells of the brain, including neurons. Neurons are polarized cells that are well-known to repress the translation of specific physiologically important mRNAs until these mRNAs are properly localized to projections [10]. Paving the way for future work, a very limited number of studies have shown using neurons that NMD can occur in projections, where FMRP is present [11–14].

In this review, we first overview the current understanding of NMD and FMRP in non-polarized cells. Since studies of NMD in polarized cells become critical to realistically examine the role of NMD — and FMRP — in the brain, we next highlight what is currently known about FMRP and NMD in neurons. We additionally present differences between non-polarized and polarized cells that might alter FMRP and NMD functions in the two cell types. We then mine existing data available in the literature that inform on the spatial metabolism of NMD and/or FMRP targets, i.e., FMRP-bound mRNAs, in the axon and soma of mouse retinal ganglion cells and hippocampal neurons. Results reveal that polarized neurons deriving from mouse brain, relative to polarized neurons differentiated in culture, have the highest capacity to localize to the axon and dendrites those NMD targets that are also directly bound by FMRP. We envision the coupling of FMRP and NMD functions in polarized cells as a means to provide a constructive burst in protein synthesis followed by mRNA clearance by NMD at cellular foci that are distal from the nucleus. We place these findings in the context of normal and, by inference, Fragile X Syndrome (FXS) neurobiology, the latter of which is characterized by FMRP deficiency that results in intellectual disability and autism.

### Mechanisms and subcellular localization of NMD in non-polarized cells

**Fig. 1:**
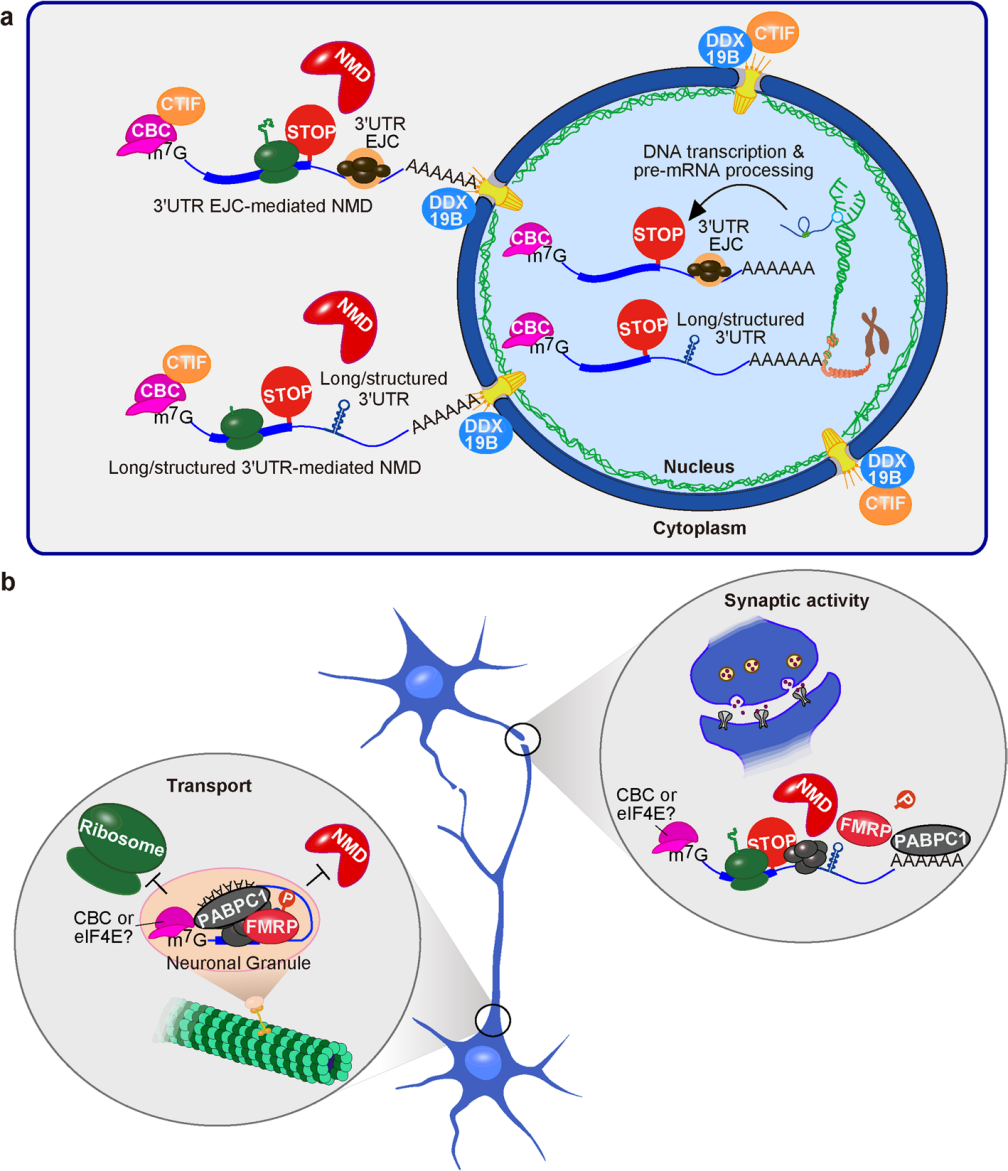
Models for NMD. **a** In non-polarized cells, the pioneer round of translation, i.e., the translation of newly made CBC-bound mRNAs, and 3′UTR EJC-mediated NMD, can take place either on the cytoplasmic side of the nuclear envelope (shown) or after disassociating with the nuclear envelop (no shown). Generally, those 3′UTR EJC-mediated NMD targets that escape decay are remodeled to contain eIF4E at their 5′ cap, lose any remaining EJCs, and become immune to further NMD (not shown), unless they continue to undergo NMD mediated by a long and/or structured 3′UTR. While the decay of newly made CBC-bound mRNAs whose NMD is triggered by a long and/or structured 3′UTR can also occur on the cytoplasmic side of the nuclear envelope, once remodeled to eIF4E-bound mRNAs, they continue to undergo NMD, presumably in the cytosol (not shown). Note that while NMD targets are generally bound by FMRP, the FMRP-mediated block in translation is incomplete since FMRP binding is not 100% efficient. FMRP is not shown. Red pacman, NMD decay machinery; CBC, CBP80 − CBP20 at the 5′ m^7^G cap of newly made mRNAs; green balls, 80S ribosome with nascent peptide; STOP, translation termination codon; EJC, exon-junction complex. **b** In polarized cells, the translation of at least a fraction of newly made mRNAs appears to be inhibited until the mRNA is properly transported in granules to a distal projection. Where in the cell and when, if at all, during transport and localization the CBC is replaced by eIF4E remains to be determined. Based on work using non-polarized cells, FMRP binding to target mRNAs, including NMD targets, requires its direct interaction with PABPC1, which is bound to the poly(A) tail of the mRNA. According to this model, once localized to a distal projection, FMRP is removed from the NMD target by the dephosphorylation of FMRP, allowing translation and, as a consequence, decay by NMD. The step(s) at which translation is inhibited prior to granule localization remains to be defined

Studies of non-polarized mammalian cells have demonstrated that the translation of newly synthesized CBP80—CBP20 (CBC)-bound mRNAs [15] begins for many mRNAs on the cytoplasmic side of the nuclear envelope within one minute after emerging from the nuclear pore into the cytoplasm [16, 17]. Restricting the pioneer round of translation, which we have defined as the translation of CBC-bound mRNA [15], to newly made mRNAs that maintain a physical association with the nucleus in which they were generated [18] has been reported to involve tethering the CBC translation initiation factor CTIF, which binds CBP80, to the perinuclear region by DDX19B (Fig. 1a) [17]. DDX19B is a DEAD-box RNA helicase that localizes to the cytoplasmic side of the nuclear pore and is activated by nucleoporins [19, 20]. Yet to be resolved is the relationship between CTIF, which shares domains with eukaryotic translation initiation factor eIF4G1, and eIF4G1 itself, each of which independently support the pioneer round of translation by bridging the CBC and the translation initiation complex [21, 22].

Without DDX19B-mediated handing-off of CTIF to CBC-bound mRNA, the pioneer round of translation occurs after dissociation from the nuclear envelop [17], as exemplified by glutathione peroxidase 1 (*GPx1*) mRNA, a physiological NMD target [23]. However, the intracellular site of the *GPx1* mRNA pioneer round of translation can be moved to the perinuclear space by overexpressing the RNA-binding protein SRSF1 [24]. It is now becoming clear that RNA-binding proteins — in particular, those that bind to mRNA 3′-untranslated regions (3′UTRs), can influence the cytoplasmic compartment in which an mRNA is translated [25, 26].

Whether in the perinuclear space or nucleus-distal regions of the cytoplasm, CBC-bound mRNAs harbor generic RNA-binding proteins needed to undergo immediate and efficient NMD, should the specific requirements for NMD have been met. These proteins include CBP80 itself and a post-splicing exon-junction complex (EJC) [27, 28] (Fig. 1a). The 3′UTR EJC-mediated NMD of CBC-bound mRNAs occurs when the process of translation fails to remove one or more EJCs that reside downstream of the site of translation termination. This occurs when translation terminates more than 50 − 55-nucleotides upstream of a splicing-generated exon − exon junction, upstream of which an EJC has been deposited in a sequence-independent but position-dependent mechanism [29]. Subsequently, a choreographed series of protein − protein rearrangements takes place on the NMD target [27, 30, 31]. These steps include recruitment and phosphorylation of the key NMD factor UPF1, which is an ATP-dependent RNA helicase. In fact, the preferential co-immunoprecipitation (co-IP) of an mRNA with p-UPF1 provides an experimental means to identify it as a physiologic NMD target [3, 32, 33]. Phosphorylated UPF1 (p-UPF1) binds to eIF3 of a 48S pre-initiation complex bound to the NMD target’s AUG initiation codon to repress further rounds of translation initiation [34]. Additionally, p-UPF1 recruits mRNA degradative activities [35, 36] and removes proteins from the NMD target to facilitate efficient mRNA degradation [37]. As noted above, NMD is rarely, if ever, 100% efficient [38, 39], and the CBC on the fraction of newly made mRNAs that escapes 3′UTR-EJC-mediated NMD is replaced by another eukaryotic translation initiation factor, eIF4E [28], which typifies the bulk of cellular mRNAs in the steady state [40]. For NMD and non-NMD targets, the replacement of the CBC by eIF4E occurs when importin ɑ, which remains associated with the nuclear localization sequences of CBP80 during the export of CBC-bound mRNAs from the nucleus to the cytoplasm, binds the karyopherin importin β in the cytoplasm; as a consequence, 5′ cap-bound CBC is loosened so that eIF4E can take its place [41, 42]. Notably, translation is not required for replacement of the CBC by eIF4E [42]. By the time eIF4E replaces the CBC, EJCs have largely been displaced by, e.g., the pioneer round of translation, and are no longer detectable on mRNAs [28].

NMD can also occur when translation terminates upstream of a long and/or structured 3′UTR that lacks an EJC [8, 43] (Fig. 1a). Such a 3′UTR is thought to enhance the probability of UPF1 binding downstream of the translation termination event, including at a normal translation termination codon, on both CBC-bound and eIF4E-bound mRNAs [44, 45]. In this mechanism, the targeting of eIF4E-bound mRNA for NMD is evident since tethering UPF1 downstream of a normal termination codon triggers the NMD of both CBC-bound and eIF4E-bound mRNA [46]. Additionally, the suppression of eIF4E-dependent translation also inhibits the NMD of mRNAs with a long and/or structured 3′UTR [44].

How other cap-binding proteins that support translation may interface with NMD remains largely unexplored. These cap-binding proteins include eIF3d [47], which in MDA-MB-231 breast cancer cells supports ~ 25% of the translatome together with the eIF4G1-related protein DAP5 [48], and LARP1, which binds to mRNAs that harbor in their 5′UTR a so-called 5′-terminal oligopyrimidine tract [49]. Moreover, NMD has yet to be explored for those non-polarized cells having high levels of nuclear eIF4E, e.g., high-eIF4E primary leukemia samples or U2OS cells overexpressing FLAG-tagged eIF4E, in which specific nuclear mRNAs acquire eIF4E at their 5′ cap [50].

### FMRP represses NMD

FMRP is an RNA-binding protein that has been shown to repress steady-state mRNA translation in both non-polarized and polarized cells [4, 10, 51, 52], yet apparently by different mechanisms (see below). In addition to associating with eIF4E-bound mRNAs [53], data for non-polarized cells indicate that FMRP can also associate with CBC-bound mRNAs [3]. This became apparent with the finding that FMRP interacts directly with UPF1 and p-UPF1, which recruit FMRP to and/or stabilize FMRP on NMD targets so as to inhibit their degradation by NMD [3]. FMRP can also bind directly to NMD targets, as it does to non-NMD targets, at GC-rich and/or structured sequences, which are sequences that also typify UPF1- and p-UPF1-binding sites in NMD targets [[4] and references therein]. Importantly, since NMD occurs in non-polarized cells, and NMD requires translation, it follows that the FMRP-mediated inhibition of translation is incomplete in these cells. As shown using non-polarized cells, for FMRP to inhibit the translation and decay of NMD targets (and also non-NMD targets), FMRP must also bind directly to poly(A)-binding protein C1 (PABPC1) at the mRNA poly(A) tail, possibly resulting in mRNA sequestration together with known mRNA granule constituents [4].

In agreement with the observation that FMRP co-localizes with PABPC1 and granule constituents in polarized, i.e., differentiated, human SH-SY5Y neuroblastoma cells [4], we propose the existence of a translationally repressed granule — an NMD-silenced complex — that contains FMRP-bound NMD targets, protected from mRNA decay and transported to neuronal projections (Fig. 1b). Consistent with this idea, granules of this type in polarized cells regulate gene expression by mediating directed intracellular transport of the encoded mRNA followed by cell site-specific granule dissolution that allows the mRNA to undergo localized translation [54, 55]. Such regulation typifies numerous polarized cells, such as neurons, retinal ganglion cells, and radial glial cells [56, 57].

### FMRP functions in neurons: transport of translationally repressed mRNAs and microtubule integrity

Neurons exemplify highly polarized cells, possessing morphologically and functionally distinct projections, i.e., an axon and dendrites, extending from the cell body, i.e., the soma. There are a number of differences between polarized and non-polarized cells that are too numerous to detail here. For example, the microtubule cytoskeleton, which is critical for the polarized shape, intracellular order, and motility of neurons, differentiates polarized cells from non-polarized cells. In polarized cells, dynamic microtubules surround the entire neuronal-cell nucleus, and stable microtubules extend from the distal part of the nuclear envelope through the soma to the extremities of the projections [58]. FMRP is enriched in both axons and dendrites [59], and FMRP-bound mRNAs in cytoplasmic granules move through the soma along microtubules to their distal sites of translational activation [60]. Using cultured hippocampal neurons isolated from mice, high-resolution fluorescent microscopy has allowed visualizing the movement of FMRP-containing granules through the soma and the subsequent synapse activity-dependent translational activation of the constituent mRNAs in dendrites and dendritic spines [59–62]. Similarly, imaging studies of radial glial cells from control and *Fmr1*-KO embryonic mouse brain in which the gene encoding FMRP has been inactivated, demonstrated that the transport of at least some FMRP-bound mRNAs from the cell body to the endfeet, which are button-like terminals called “boutons,” is an FMRP-mediated mechanism [63]. Moreover, as visualized by tracking reporter mRNA particle dynamics, the C-terminus of FMRP is required when the group I metabotropic glutamate receptor (mGluR)-dependent synaptic activation augments the microtubule-mediated transport of FMRP- and reporter mRNA-containing granules to dendrites via kinesin light chains, thereby regulating local mRNA translation to facilitate normal synaptic maturation [60]. Whether the interaction between FMRP and kinesin is direct remains unknown.

Additionally, FMRP itself functions in microtubule integrity. For example, FMRP binds and regulates mRNAs encoding microtubule-associated protein 1B (MAP1B) [59, 64, 65] and microtubule-associated protein tau (MAPT) [66], both of which are critical for microtubule organization and neuronal polarization [67]. For these and other reasons, *Fmr1*-KO mouse neurons manifest mis-regulated microtubule stability and abnormal dendritic filopodia-spine morphology, mimicking the FXS phenotype [60, 64].

### Functional interactions between FMRP and NMD in neurons

Recent transcriptome analyses using non-polarized human SH-SY5Y neuroblastoma cells revealed that FMRP associates with hundreds of cellular mRNAs, including NMD targets, at GC-rich and/or structured sequences within their transcribed body [4]. FMRP binding to mRNA targets is promoted by FMRP directly interacting with PABPC1 that is bound to the mRNA poly(A) tail [4]. In these cells, NMD targets are enriched among FMRP-bound mRNAs, and many FMRP-bound NMD targets play important roles in synaptic signaling [3, 33]. Microtubule-associated proteins encoded by *Map1b* and *Mapt* mRNAs exemplify FMRP-bound physiologic NMD targets in N2A cells that are destabilized in cultured neurons derived from *Fmr1*-KO postnatal day 1 (P1) cortex and, thus, are likely to be NMD targets in mouse brain [33]. These mRNAs also localize to and are translated at dendrites of mouse cortical neurons, hippocampal neurons, and mouse P19 embryonic carcinoma cells that were differentiated to neurons [64, 68, 69]. NMD also degrades other FMRP-bound NMD targets that localize to and are translated at in dendritic projections, such as mRNA encoding activity-regulated cytoskeleton-associated protein (ARC), to regulate synaptic plasticity and cognitive function in mouse brain [12, 65]. In *Drosophila* laminar neurons, like FMRP [70], NMD plays a role in maintaining normal synapse formation and neurotransmission [71].

It remains unknown what differences between polarized and non-polarized cells explain how the translation of FMRP-bound NMD targets in polarized cells and/or EJC removal is inhibited until the mRNAs are properly localized. If translation is inhibited, then in theory the translation of CBC-bound mRNA, eIF4E-bound mRNA, or both could be inhibited (see below). Key differences between polarized and non-polarized cells may include a distinct physical coupling of the nucleus with the cytoskeleton, specialized cytoskeletal or motor proteins, and/or one or more RNA-binding proteins that influence the fate of newly made FMRP-bound NMD targets. The latter concept is particularly attractive since computational analyses of human and mouse brains have revealed the presence of 3′UTRs generated by alternative cleavage and polyadenylation (APA) that are much longer than in non-neuronal cells [72]. In addition to influencing long and/or structured 3′UTR-mediated NMD, APA may regulate mRNA translation, mRNA localization, and interactions of the encoded protein with other proteins without changing the level of mRNA expression [73, 74]. Illustrating the importance of APA events that generate longer 3′UTRs, which are common during central nervous system development and neuronal differentiation, are the many neurological disorders that have been linked to misregulated APA [75]. In the case of NMD targets, longer 3′UTRs would conceivably allow for more FMRP recruitment/stabilization via more UPF1 and p-UPF1 binding, and thus, more efficient translational repression.

### FMRP-bound NMD targets most efficiently localize to neuronal projections

Consistent with FMRP enrichment in dendrites and axons [59], transcriptomic analyses of RNAs extracted from the soma and, separately, from the dendrites of hippocampal CA1 pyramidal neurons, or from the soma and, separately, from the axon of retinal ganglion cells, showed that FMRP targets are abundant in either projection, respectively [13, 14]. However, how NMD targets localize to the projection of these neuronal cells with respect to their binding to FMRP is unclear. Our focus on FMRP-bound mRNAs that are NMD targets is important given the remarkable contributions of NMD to synaptic signaling and neuronal transmission [3, 33], and thus to neurobiology in health and disease [7, 76].

To date, a sufficient number of mRNAs have been localized within retinal ganglion cells [14] and hippocampal neurons [13] from mice for us to query where in these neurons NMD targets and FMRP targets are enriched. We analyzed the 1027 high-confidence NMD targets that we had defined for mouse N2A neuroblastoma cells and shown to be directly or indirectly bound by p-UPF1 using RIP-seq, i.e., anti-p-UPF1 IP of cell lysates followed by RNA sequencing [33]. Notably, among these NMD targets, all tested were found to be destabilized in *Fmr1*-KO P1 cortical neurons [33], reinforcing the idea that FMRP generally stabilizes NMD targets, which it binds via UPF1 and p-UPF1, if not also directly [3]. We also defined 5733 cellular transcripts found to be directly bound by FMRP after anti-FMRP or anti-GFP-FMRP UV Cross-Linking IP (CLIP)-seq (i.e., regardless of FMRP binding to UPF1 and p-UPF1) of (i) mouse cerebral cortex, hippocampus, and cerebellum [77]; (ii) mouse hippocampal CA1 pyramidal neurons or cerebellar granules cells, using excitatory neuron-restricted expression of GFP-tagged FMRP in lysates of hippocampus or cerebellum, respectively [78]; or (iii) total mouse brain, using polysome fractions deriving from mouse brain lysates [79]. Notably, some of these NMD targets and FMRP-bound mRNAs have been mis-annotated as long noncoding RNAs (mouse GENCODE database version M32; https://www.gencodegenes.org/) [80, 81].

**Fig. 2:**
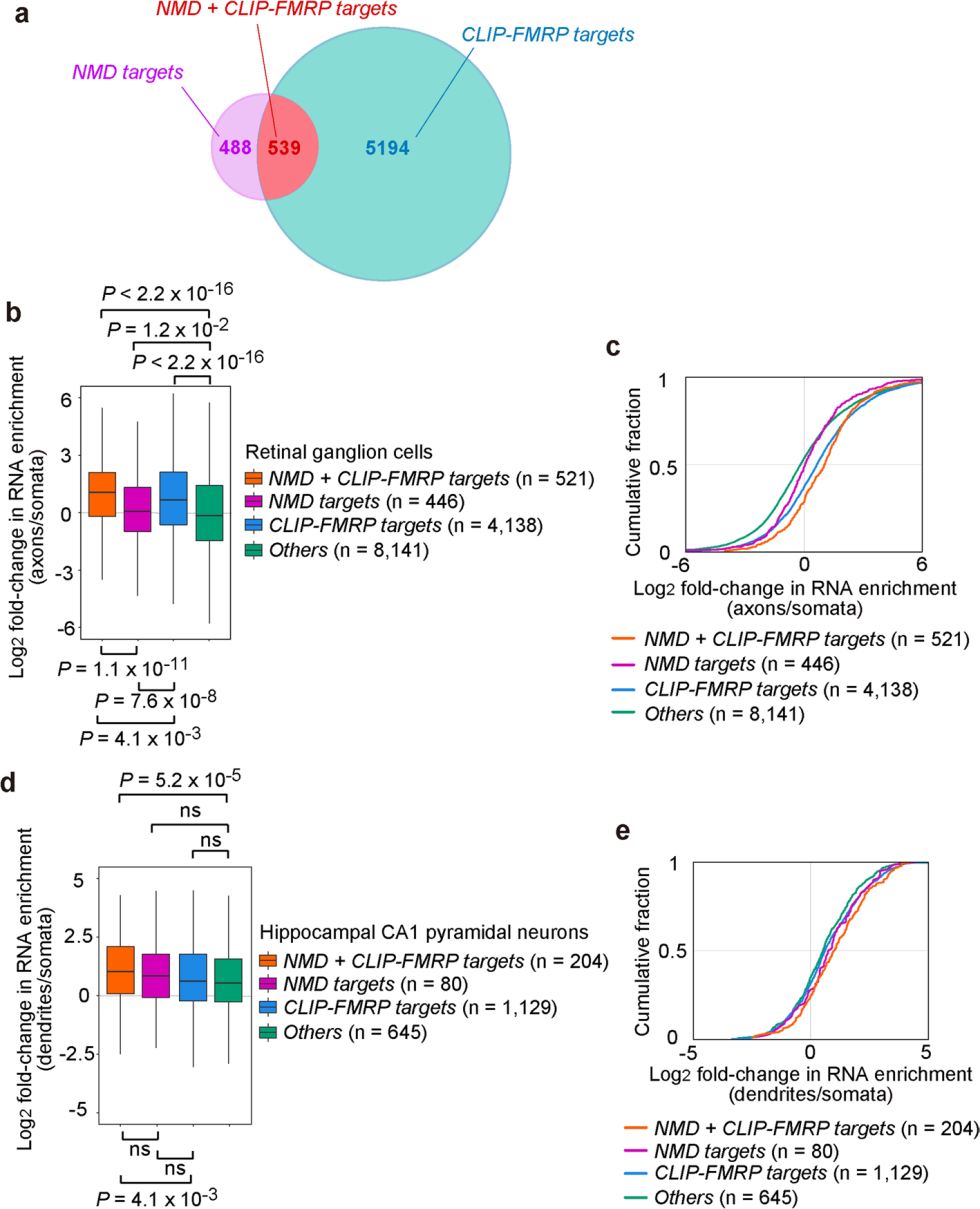
Overlap between NMD and FMRP targets in neuronal cells or tissues, and subcellular localization of NMD and FMRP targets in neuronal cells. **a** Venn diagram showing the overlap between the 1027 NMD targets defined using mouse N2A neuroblastoma cells [33], and the 5733 FMRP targets defined using total mouse cerebral cortex [77], mouse hippocampus [77], mouse cerebellum [77], mouse hippocampal CA1 pyramidal neurons [78], mouse cerebellar granule cells [78], or mouse whole brain polysome fractions [79]. The statistical significance of the overlap between “*NMD targets*” and “*CLIP-FMRP targets*,” i.e., “*NMD* + *CLIP-FMRP targets*,” in the pool of 21,565 protein-coding genes derived from the mouse GENCODE M32 database (https://www.gencodegenes.org/) was calculated using Fisher’s exact test (*P* < 2.2 × 10.^−16^, odds ratio = 3.26, 95% confidence interval 2.87–3.71) in R version 3.6.3 [82]. **b** Box and whisker plots showing log_2_ fold-changes in RNA enrichment (axons/somata) using mRNAs defined in **a**, after removing outliers, and data deriving from mouse retinal ganglion cells [14]. Outliers were identified, and plots were generated using the ggplot2 program (https://ggplot2.tidyverse.org/index.html) in Tidyverse package (https://www.tidyverse.org/) and R version 3.6.3. The top, middle, and bottom of each box, represent, respectively, the first, second, or third quartile of the dataset. *n*, number of mRNAs identified in **a** that existed in the dataset derived by Jung et al. [14] and was used for the statistical analysis. P values were calculated using the two-sided Wilcoxon rank-sum test using R version 3.6.3 for the full data-set, including outliers. **c** Cumulative fraction of log_2_ fold-change RNA enrichment (axons/somata) using mRNAs defined in **a**, and data deriving from mouse retinal ganglion cells [14]. **d** As in **b**, box and whisker plots showing log_2_ fold-changes in RNA enrichment (dendrites/somata) using mRNAs defined in **a**, after removing outliers, and data deriving from mouse hippocampal CA1 pyramidal neurons [13]. P values were calculated as in **b**. **e** As in **c**, but using data deriving from mouse hippocampal CA1 pyramidal neurons [13]

Supporting a mechanistic connection between FMRP and NMD [3, 33], in silico analyses of these datasets revealed that the overlap between NMD targets and FMRP targets was significantly larger than expected by chance (*P* < 2.2 × 10^−16^, Fisher’s exact test): 539 of these 1027 NMD targets were among the CLIP-FMRP targets (Fig. 2a). However, there are several caveats to our computational analyses that could explain why only 539 NMD targets identified for mouse N2A cells overlap with the 5733 CLIP-FMRP targets that were identified using six different mouse brain-derived transcriptomes. These caveats do not invalidate our results but should be considered and are as follows.

First, NMD targets were defined using N2A cells and two verified criteria, namely, (i) upregulation upon UPF1 downregulation, and (ii) co-IP with p-UPF1 [33], each with stringent thresholds that undoubtedly do not yield a comprehensive list of NMD targets in these cells. Second, the number of NMD targets deriving from mouse N2A cells is likely to be a significant underestimate of NMD targets in mouse brain cells if recent analyses of the translational landscape in human prenatal and adult brains [83] can be extrapolated to mouse brain. The human brain analyses employed RNA deep sequencing of ribosome-protected RNA fragments (ribo-seq) to identify 38,187 actively translated small uORFs, encoded by 8278 genes, that were either out-of-frame or did not overlap with the main ORF [83]. Considering that a uORF typically ends with a termination codon located > 50 − 55 nucleotides upstream of an exon − exon junction, which is a hallmark of NMD targets (see above), there are likely many more NMD targets not only in human brain but also in mouse brain that have yet to be defined. Third, the 5733 FMRP-bound targets were defined using CLIP [77–79], which may underestimate the total number of mRNAs directly bound by FMRP due to crosslinking bias during CLIP [84, 85]: UV preferentially generates crosslinks between pyrimidines in single-stranded RNA and the side chains of aromatic amino acids; however, FMRP prefers to bind G-rich and structured sequences, and the FMRP RNA-binding RGG box lacks aromatic amino acids [4]. Fourth, the recruitment and/or stabilization of FMRP on mRNAs by UPF1 and p-UPF1, and possibly other RNA-binding proteins, e.g., the FMRP paralog FXR2 [86] or the m^6^A reader YTHDF1 [87] (see below), may not involve direct binding of FMRP to mRNA and therefore may be undetectable using CLIP. Fifth, that all molecules of any particular NMD target or other FMRP target are actually bound by FMRP at any one time is unlikely.

With those caveats in place, we first defined four different cellular mRNA categories: (i) “*NMD* + *CLIP-FMRP targets*,” i.e., mRNAs that are high-confidence NMD targets and that CLIP with FMRP; (ii) “*NMD targets*,” i.e., mRNAs that are high-confidence NMD targets that did not detectably CLIP with FMRP but are associated with FMRP via UPF1/p-UPF1 [3, 33]; (iii) “*CLIP-FMRP targets*,” i.e., mRNAs that were identified using CLIP as FMRP targets but are not among the high-confidence NMD targets; and (iv) “*Others*,” i.e., RNAs that are not among groups (i) − (iii). Results showed that the enrichment in the axon compared to soma for retinal ganglion-cell RNAs [14] is: “*NMD* + *CLIP-FMRP targets*” > “*CLIP-FMRP targets*” > “*NMD targets*” > “*Others*” (Fig. 2b, c). Quantitations revealed that the ratio in axons:somata for each of the four mRNA categories is, respectively, ~ 72%:28%, ~ 63%:37%, ~ 52%:48%, and ~ 47%:53% (Fig. 2b, c). A more subtle and slightly different polarized hierarchy was obtained for mRNAs expressed in hippocampal CA1 pyramidal neurons [13], i.e., enrichment in dendrites relative to soma of “*NMD* + *CLIP-FMRP targets*” > “*NMD targets*” > “*CLIP-FMRP targets*” > “*Others*” (Fig. 2d, e).

As noted above, Hale et al. [13] found that their own definition of CLIP-FMRP targets, which includes mRNAs that we have defined as NMD targets [33], were enriched in the dendrites of CA1 hippocampal neurons. Our findings refine their conclusion by showing that, as a rule, FMRP-bound NMD targets are preferentially enriched in axons and dendrites relative to somata when compared to FMRP targets that are not high-confidence NMD targets or NMD targets that do not CLIP with FMRP. In other words, our results suggest that both direct binding of FMRP to cellular mRNAs and binding of FMRP via UPF1 and p-UPF1 to physiologic NMD targets promote mRNA localization to neuronal projections in a cumulative manner. Nonetheless, relative to UPF1- and p-UPF1-mediated binding, direct binding by FMRP to target mRNAs is a bigger contributor to the enrichment of mRNAs in axons. Conversely, while statistical analyses are less conclusive, it appears that, relative to direct binding, UPF1- and p-UPF1-mediated binding of FMRP to mRNAs is a bigger contributor to the enrichment of mRNAs in dendrites.

### In *Fmr1*-KO neurons, other factors can localize mRNAs normally bound by FMRP

**Fig. 3:**
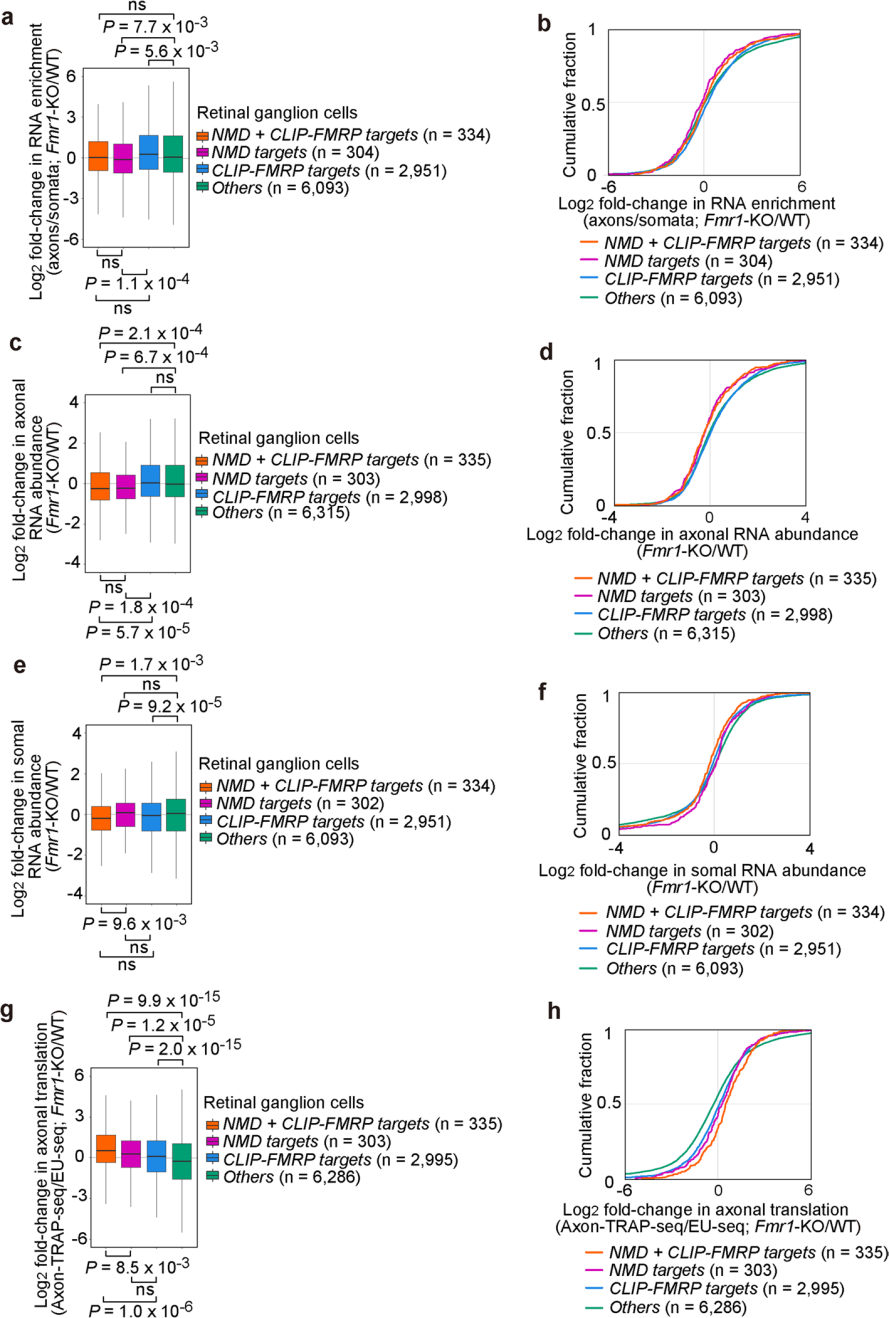
FMRP-dependent localization and translation of NMD and FMRP targets in the axon relative to soma of mouse retinal ganglion cells. **a** Box and whisker plots as in Fig. 2b using mRNAs defined in Fig. 2a, after removing outliers, and data deriving from retinal ganglion cell axon and soma of *Fmr1*-KO mice relative to WT mice [14]. *P* values were calculated as in Fig. 2b. **b** Cumulative fraction of log_2_ fold-change RNA enrichment (axons/somata) using mRNAs defined in Fig. 2a, and data deriving from retinal ganglion cell axon and soma of *Fmr1*-KO mice relative to WT mice [14]. **c** Box and whisker plots as in Fig. 2b using mRNAs defined in Fig. 2a, after removing outliers, and data deriving from retinal ganglion cell axons of *Fmr1*-KO mice relative to WT mice [14]. P values were calculated as in Fig. 2b. **d** Cumulative fraction of log_2_ fold-change axonal RNA enrichment using mRNAs defined in Fig. 2a, and data deriving from retinal ganglion cell axons of *Fmr1*-KO mice relative to WT mice [14]. **e** As in **c**, but using data deriving from retinal ganglion cell somata of *Fmr1*-KO mice relative to WT mice [14]. P values were calculated as in Fig. 2b. **f** As in **d**, but using data deriving from retinal ganglion cell somata of *Fmr1*-KO mice relative to WT mice [14]. **g** As in **a**, box and whisker plots showing log_2_ fold-changes in axonal translation (Axon-TRAP-seq normalized to mRNA abundance determined using EU-seq) for mRNAs defined in Fig. 2a, after removing outliers, and data deriving from retinal ganglion cell axons of *Fmr1-*KO mice relative to WT mice [14]. P values were calculated as in Fig. 2b. **h** Cumulative fraction of log_2_ fold-change in axonal translation, (Axon-TRAP-seq normalized by EU-seq) using mRNAs defined in Fig. 2a, and data deriving from retinal ganglion cell axons of *Fmr1-*KO mice relative to WT mice [14]

We next examined ribosome-bound mRNAs [14] to determine the consequence of FMRP loss on mRNA localization and translation. In agreement with the conclusion drawn by Hale and co-workers using CA1 hippocampal neurons [13], our comparison of data deriving from WT and *Fmr1*-KO mice [14] does not support a major need for FMRP in the spatial distribution of FMRP-bound mRNAs in retinal ganglion cells. Relative to “*Others*,” *Fmr1*-KO modestly increased “*CLIP-FMRP targets*” in axons/somata (Fig. 3a, b). In contrast, *Fmr1*-KO modestly decreased “*NMD targets*” in axons/somata (Fig. 3a, b). Rather than a change in NMD target transport, this observation is likely due to a decreased abundance of “*NMD targets*” in the axon (Fig. 3c, d) but not soma (Fig. 3e, f) of *Fmr1*-KO retinal ganglion cells, which could reflect localized hyperactivation of NMD [3, 33]. The intracellular distribution of “*NMD* + *CLIP-FMRP targets*” was largely similar to that of “*Others*,” suggesting that mild contributions of direct FMRP binding and being an NMD target, i.e., FMRP binding via UPF1 and p-UPF1, offset one another (Fig. 3a, b).

Despite these findings, FMRP-bound mRNAs are enriched in neuronal projections (Fig. 2b–e), and FMRP directly promotes the axon localization of at least some FMRP-bound mRNAs [60, 63]. It follows that, for the majority of NMD and non-NMD targets, the loss of FMRP-binding may allow for the binding of another protein that likewise promotes mRNA localization to axons. In behavioral studies that measured hyperactivity and severe learning and memory impairments, one protein that appears to be able to substitute for FMRP function in *Fmr1*-KO mice is FXR2 [88–90], a FMRP paralog with 60% amino acid identity to FMRP and similar functional-domain structure [86]. In support of FMRP and FXR2 manifesting overlapping functions, relative to WT mice, the degree of mGluR-dependent long-term depression (mGluR-LTD) in the hippocampus was exacerbated in *Fmr1*-KO mice in which the *Fxr2* gene was also knocked out [91]. These findings suggest that, when FMRP is deficient, FMRP-dependent localization of FMRP-bound mRNAs may be mediated by FXR2. In further support of this possibility, the RNA-binding properties of the FMRP and FXR2 KH2 domains are very similar [92]. Moreover, for NMD targets, FXR2, like FMRP, co-immunoprecipitates with p-UPF1 in an RNase I-resistant manner using HEK293T-cell lysates [3]. However, since FXR2 lacks an RGG domain [86], if and how FXR2 could be recruited to non-NMD targets that FMRP binds via G-quadruplexes remains unclear.

FMRP and FXR2 are certainly not the only cellular determinants that facilitate localized translation of FMRP-bound mRNAs [14, 93]. For example, a case could be made for the hnRNP protein TDP-43 as an inhibitor of the FMRP-bound NMD targets in polarized neuronal cells until the targets are properly localized in distal projections. First, purified *D. melanogaster* FMRP and *D. melanogaster* TDP-43 interact in vitro, and exogenously expressed human TDP-43 assembles with endogenous FMRP in *D. melanogaster* neurons [94]. Consistent with these findings, FMRP and TDP-43 co-immunoprecipitate from lysates of HEK293T cells and mouse hippocampal neurons [94, 95], and thus possibly function together in the transport and translational repression of NMD targets (as well as other FMRP-bound mRNAs) in polarized cells. In support of this possibility, FMRP and TDP-43 share mRNA targets in neurons [95, 96] where, again, some but not all may be NMD targets. Second, TDP-43 stands out as a key regulator of mRNP transport through the somata to the axons of retinal ganglion cells, after which translation occurs [14]. Third, TDP-43 is needed for the transport of mRNPs into axons [97, 98] and dendritic spines [99] and, using real-time visualization of mRNA transport and translation, TDP-43 inhibits mRNA translation within actively transporting mRNPs until they reach dendritic spines [99]. Fourth, biochemical approaches and super-resolution microscopy have verified that TDP-43 constitutes neuronal mRNP granules and manifests post-synaptic localization [100]. These studies also showed that the activity-dependent dynamics of mRNP granules involve their disassembly, release of the component mRNAs, and the activation of local protein synthesis, all of which are impaired in cellular, animal, and human models of TDP-43 proteinopathy [100].

### FMRP represses most efficiently the translation of FMRP-bound NMD targets

Since NMD is a translation-dependent process, we also mined Axon-Translating Ribosome Affinity Purification-seq (Axon-TRAP-seq) data to determine the relative abundance of NMD targets in the axons of WT and *Fmr1*-KO mice [14]. Axon-TRAP-seq of mouse retinal ganglion cells showed that the axonal translation of FMRP-regulated mRNAs defined by Jung et al. [14], after normalization to total axonal mRNA abundance using 5′-ethynyl uridine sequencing (EU-seq), was significantly enhanced in *Fmr1*-KO mice relative to WT mice [14]. Results from our refined analyses showed that the axonal translation in retinal ganglion cells of *Fmr1*-KO mice relative to WT mice is: “*NMD* + *CLIP-FMRP targets*” > “*NMD targets*” > “*CLIP-FMRP targets*” > “*Others*” (Fig. 3g, h). Thus, similar to our conclusions for the localization of FMRP-bound mRNAs and NMD targets to neuronal projections, direct FMRP binding, and FMRP binding via UPF1 and p-UPF1, each contribute to FMRP-mediated translational repression in a cumulative manner. However, unlike the FMRP being dispensable for the localization of FMRP-bound mRNAs and NMD targets to neuronal projections (Fig. 3a, b), FMRP orchestrates axon-localized translational repression of both NMD and non-NMD targets in a way that cannot be fully compensated for by other cellular constituents, such as FXR2, when FMRP is lost.

Importantly, the increased translation of “*NMD targets*” in axons of *Fmr1*-KO neurons (Fig. 3g, h) correlates with a decrease in mRNA abundance (Fig. 3c, d). Thus, we hypothesize that for NMD targets, eventual release from FMRP-mediated translational repression in axons is accompanied by protein production during what presumably, at least in part, targets the translation of CBC-bound mRNA and, as a consequence, rapid mRNA degradation by NMD. This ensures a burst of localized protein expression. It follows that the pioneer rounds of translation that trigger NMD at least sometimes occur in neuronal projections — a conclusion that is supported by a large body of literature as discussed below.

### Evidence for CBC-bound mRNA translation at polarized-cell projections, including neurons

Evidence that the inhibition of translation can occur prior to the replacement of CBC by eIF4E at the 5′-mRNA cap in polarized cells derives from demonstrations that oskar mRNA in the developing *Drosophila melanogaster* oocyte fails to undergo the “first round of translation” until the mRNA reaches the posterior pole [101]. As additional evidence, components of the pioneer translation initiation complex, including CBP80 and proteins that constitute EJCs, are concentrated at polarized-cell projections. For example, the dendrites of mature rat hippocampal neurons immunostain with antibodies to CBP80 as well as the auxiliary EJC constituent CASC3 (also called Barentz) in RNA granule-like puncta [102]. CBP80-positive immunoreactivity at puncta that anneal with oligo(dT), i.e., that are interpreted to contain poly(A)^+^ mRNAs, have been characterized in neuronal projections of dopaminergic cells derived from human iPSC lines and in the branched projections in SH-SY5Y cells cultured sequentially in retinoic acid and brain-derived neurotrophic factor (BDNF) to promote their polarization [103]. Notably, CBP80 puncta in polarized SH-SY5Y cells, unlike eIF4E puncta, were found distributed along anti-tubulin-staining fibers, were enriched in the core EJC constituents RBM8A and eIF4A3, and were insensitive to the translational inhibitor cycloheximide [103]. These results suggest that these CBP80 puncta constituted newly made translationally repressed mRNPs being transported along microtubules from the nucleus to neuronal projections and had yet to undergo the pioneer round of translation [103]. This agrees with the co-localization of FMRP with the G3BP1 granule constituent in the soma of SH-SY5Y cells that were differentiated to polarized cells using retinoic acid followed by BDNF [4]. Finally, other studies have demonstrated localization of the core EJC constituents eIF4A3, RBM8A, and MAGOH as well as the auxiliary EJC constituent CASC3 to the dendritic extensions of neuronal somata [11, 104–106].

Studies of specific NMD targets also support the idea that their translation can be inhibited prior to the loss of EJCs, if not also prior to the loss of 5′ cap-bound CBC in neuronal projections. Newly made *Arc* mRNA is targeted for NMD due to its 3′UTR EJC, which contains eIF4A3 [29]. eIF4A3 knockdown in cultured rat neurons treated with BDNF increased *Arc* mRNA levels in both somata and dendrites and increased ARC protein levels not only in somata and dendrites but also at synapses [11]. The accumulation of ARC protein at synapses was accompanied by increased excitatory postsynaptic strength and increased surface abundance of the AMPA receptor GluR1 at putative postsynaptic sites [11]. These findings are consistent with the role of eIF4A3 in mediating the NMD of *Arc* mRNA, and the translational silencing of some (but notably not all; see below) *Arc* mRNA molecules when in transit through the soma until undergoing the pioneer round of translation at synapses. Addressing the issue of how a short-lived burst in neuronal stimulation could support long-term memory, real-time imaging of *Arc* mRNA movement in individual neurons demonstrated that a single stimulatory event was adequate to induce synapse-localized ARC protein synthesis at so-called translational hubs, from which the resulting newly made ARC protein then fed back to induce *Arc* gene transcription [107]. Notably, some *Arc* mRNA at these translational hubs appears to be associated with eIF4E at the 5′ cap [108]. Whether these mRNAs escaped or already underwent the pioneer round of translation will determine the fraction of these eIF4E-bound mRNAs that retain their post-splicing EJCs: conceivably, sufficient time may have elapsed between the synthesis of an mRNA and its translation once localized to the hub that the CBC has already been replaced by eIF4E, since, as noted above, the replacement of CBC by eIF4E does not require translation [42]; alternatively, replacement may be actively inhibited. Another example of localized translation for which the nature of the cap-binding protein remains unknown occurs in developing mouse spinal cord: the NMD target encoding ROBO3.2, which is an axon guidance cue in commissural neurons, is translated and subsequently targeted for 3′UTR EJC-mediated NMD in response to signals that derive from the floor-plate axon termini when axons cross the spinal cord midline so as to promote axon transit across the midline [109]. For each localized NMD target, their translation and the subsequent decay would occur with the appropriate signal. One such signal would be the activity-dependent dephosphorylation of FMRP by protein phosphatase 2A (PP2A), which dissociates FMRP from the mRNA, relieving the mRNA of protection from translation and decay so as to control processes that include postsynaptic plasticity [110] (Fig. 1b).

### Evidence for alternative FMRP-containing NMD-target granules

Mounting evidence indicates that there are many types of FMRP-containing mRNA granules in neurons, some of which are translationally active, and others of which are translationally repressed [111, 112]. There is also support for the idea that translational repression in granules may be accomplished by inhibiting different steps of translation, depending on the type of granule [111, 113]. To date, combinations of DEAD-box proteins have been used to define different classes of mRNA granules in neurons [114]. Additionally, whether or not the mRNAs in RNA granules harbor CBP80 or eIF4E would indicate whether they are, respectively, blocked in the pioneer round of translation or blocked in steady-state translation, the latter of which could, but may not, occur after evading NMD. In particular, neuronal NMD targets that are bound by CBP80 or eIF4E and EJCs would be in FMRP-containing so-called “Transport granules,” which are defined as lacking 40S and 60S ribosomal subunits and, thus, blocked prior to 40S scanning to the translation initiation codon. Alternatively, NMD targets could be in FMRP-containing so-called “RNA granules,” which are defined as having both 40S and 60S ribosomal subunits and are blocked in the process of translation after 60S joining or during elongation. As an example for a mouse FMRP-bound NMD target, *Map1b* mRNA exists in polysome-stalled granules of rat hippocampal neurons [115].

A variable that may define the metabolism of FMRP-containing mRNA granules is the mechanism by which FMRP associates with mRNAs. As discussed above, while CLIP-defined FMRP binding to mRNAs, including NMD targets, is necessarily direct, RIP-defined FMRP binding to NMD targets could include direct modes of binding detected using CLIP as well as UPF1- and p-UPF1-mediated FMRP binding, the nature of which has yet to be determined. The recent report that FMRP can be recruited to m^6^A-modified mRNAs by the m^6^A reader YTHDF1, which also regulates the formation of RNA granules [87], offers yet another mechanism by which FMRP can be recruited to mRNAs in general and thus, most likely, also to NMD targets. This mechanism is activated by KCl depolarization treatment of cultured mouse cortical neurons. Prior to activation, m^6^A-containing mRNAs are translationally repressed in granules in which m^6^A-bound YTHDF1 tethers unphosphorylated FMRP, which represses translation. Activation results in FMRP phosphorylation (that, for reasons unknown, is the opposite of FMRP activation at synapses, which involves FMRP dephosphorylation) followed by FMRP loss so that condensation is now mediated by YTHDF1, which in the absence of FMRP promotes translation [87]. Notably, m^6^A-modified mRNAs, relative to unmodified mRNAs, are preferentially transported in hippocampal neurons from mice [116].

### Concluding remarks and future perspectives

Our results indicate that FMRP controls localized translation and decay of NMD and non-NMD targets. For NMD targets, the underlying mechanism partially relies on the UPF1- and p-UPF1-mediated recruitment and/or stabilization of FMRP binding in somata, which represses translation and, thus, NMD. The eventual release from translation repression and consequential activation of NMD in neuronal projections occur when FMRP dissociates, e.g., in response to synaptic activation. FMRP can also bind NMD targets and other mRNAs directly, instead of via UPF1 and p-UPF1. We hypothesize that the two mechanisms of FMRP binding have cumulative effects: In axons, the strongest effects of FMRP on translational repression (Fig. 3g, h) and on mRNA abundance, which is most likely stabilization (Fig. 3c, d), characterize “*NMD* + *CLIP-FMRP targets*”. In other words, FMRP mediates its effects most efficiently when FMRP binds directly to NMD targets, which also recruit FMRP via UPF1 and p-UPF1. Notably, it is possible if not likely that a fraction of NMD targets that happen not to be bound by FMRP undergo the pioneer round of translation and NMD in the soma, prior to localization, since pathways in cells are rarely if ever 100% efficient.

While we demonstrate that FMRP is required for efficient translational repression of “*NMD* + *CLIP-FMRP targets*,” “*NMD targets*,” and “*CLIP-FMRP targets*” (Fig. 3g, h), we also show that FMRP is not required for significant enrichment of these mRNAs in neuronal projections (Fig. 3a, b). That FMRP is not required for significant enrichment in neuronal projections contrasts conclusions drawn from previous studies using control and *Fmr1*-KO mice [59–63]. However, these previous studies were restricted to a small subset of FMRP-bound mRNAs, which our analyses indicate are not representative of the FMRP−mRNA interactome.

Notably, when we computationally mined published data from Cath.-a-differentiated (CAD) cells that had been differentiated to polarized cells in vitro, i.e., in culture [117], we obtained results that were unlike the results obtained from cells differentiated to polarized cells in vivo, i.e., in a mouse. Rather than finding that “*NMD targets*,” “*NMD* + *CLIP-FMRP targets*,” and “*CLIP-FMRP targets*” were enriched in neuronal projections, as in mouse (Fig. 2), none of the three categories of mRNAs from cells differentiated to polarized cells in vitro was enriched in neurites relative to the soma (Additional file 1: Fig. S1a,b). Thus, while these in vitro-differentiated cells manifest aspects of polarity, relative to neurons in vivo, their differentiation may not be to an extent that supports the localization of FMRP-bound mRNAs to projections.

The exact step at which the translation of NMD targets is inhibited by FMRP as they move in granules from the nucleus to their site of translational activation at the projections of polarized cells remains uncertain. As one possibility, NMD targets could be transported on polysomes that are paused during the pioneer round of translation, or after the replacement of the CBC by eIF4E, at the step of elongation or termination, consistent with the report that FMRP is found primarily on polysomes in mouse brain [79]. However, data that visualized ribosomes associated with nascent peptides using puromycylation in fixed and permeabilized hippocampal rat neurons that had been treated with emetine (to inhibit translation elongation) and puromycin (to label the C-terminus of the nascent peptide) cannot be used as an additional argument that mRNAs can be transported on polysomes to neurites [115, 118]. This is because puromycin, rather than assuredly staying localized with the ribosome-bound mRNA template, does not necessarily coincide with sites of active translation even in the presence of emetine [119]. Polysomes are indeed found throughout the length of dendrites and in dendritic spines, but where they were formed remains to be determined, and 80S monosomes, i.e., single ribosomes, appear to be much more abundant than polysomes in growing axons and growth cones [56]. As another possibility for the step at which the translation of NMD targets is inhibited, data indicate that FMRP can repress translation initiation in mouse brain [53, 65]. Thus, additional research is required to define the exact mechanisms that inhibit the translation of FMRP-bound NMD targets in transit to the projections of polarized cells, and the conditions that support these mechanisms.

### Supplementary Information

**Additional file 1. **FMRP-mediated NMD target localization in *in vitro*-differentiated mouse CAD neuronal cells fails to recapitulate FMRP-mediated NMD target localization *in vivo*, i.e. in the mouse.

**Additional file 2**. Peer review history.
