# Supplementary material for: FMRP-mediated spatial regulation of physiologic NMD targets in neuronal cells
Source: Genome Biol. 2024 Jan 23;25:31. doi: 10.1186/s13059-023-03146-x (PMC10804635; doi:10.1186/s13059-023-03146-x)
Supplement: Supplementary file 1 — Additional file 1. FMRP-mediated NMD target localization in in vitro-differentiated mouse CAD neuronal cells fails to recapitulate FMRP-mediated NMD target localization in vivo, i.e. in the mouse. [file 13059_2023_3146_MOESM1_ESM.pdf]

Additional file 1

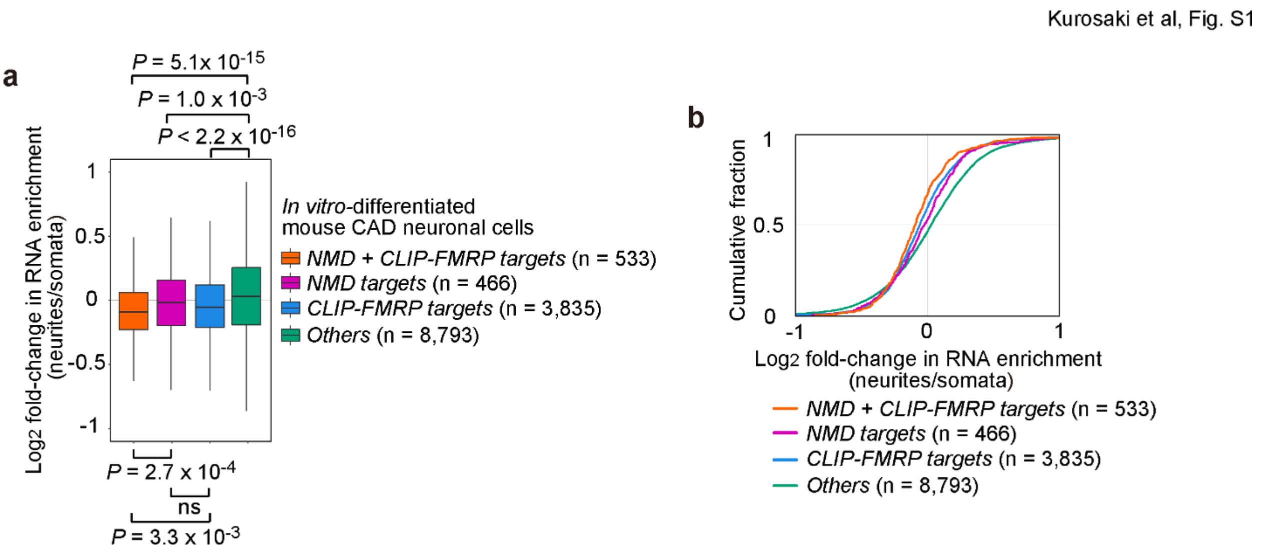

Fig. S1 FMRP-mediated NMD target localization in *in vitro*-differentiated mouse CAD neuronal cells fails to recapitulate FMRP-mediated NMD target localization *in vivo*, i.e. in the mouse

a Box and whisker plots showing $\log_2$ fold-change in RNA enrichment using mRNAs defined in **Fig. 2a,** after removing outliers, and data deriving from neurites and the soma of *in vitro*-differentiated CAD mouse neuronal cells [116]. CAD cells were induced to differentiate into a more neuronal state through the withdrawal of serum for 48 hr. Colored boxes are as in **Fig. 2b.** P values were calculated as in **Fig. 2b**.

b Cumulative fraction of $\log_2$ fold-change in RNA enrichment (neurites/somata) using mRNAs defined in **Fig. 2a,** and data deriving from neurites and the soma of *in vitro*-differentiated CAD mouse neuronal cells [116].
