# Supplementary material for: FMRP-mediated spatial regulation of physiologic NMD targets in neuronal cells
Source: Genome Biol. 2024 Jan 23;25:31. doi: 10.1186/s13059-023-03146-x (PMC10804635; doi:10.1186/s13059-023-03146-x)
Supplement: Supplementary file 2 — Additional file 2. Peer review history. [file 13059_2023_3146_MOESM2_ESM.docx]

Review History

**First round of review**

**Reviewer 1**

**Comments to author:**

This important manuscript provides evidence for a molecular model for the bursts of protein translation that are critical for neuronal function. The authors propose that a complex of FMRP and NMD proteins bound to mRNAs modulate a burst of translation at the synapse followed by RNA degradation. As the majority of NMD studies have been conducted in cell lines, it was critically important to examine its role in brain by mining existing data sets from neuronal tissues and substructures. Accordingly, the authors provide evidence for their model by mining a large number of datasets from other labs, as well as their own.

Although clever and likely true, in part, a problem with this model is that mRNAs must then be continuously supplied by the cell body to the extreme reaches of the neuronal processes. It seems much more efficient to translate an mRNA localized to a distal sight and then stop translation. It would be helpful if the authors addressed this concern. Specifically, the authors should justify why it is important to degrade the mRNA (through NMD), rather than just suppress or cease its translation.

Further points are indicated below:

1.	Some proteins described in the text are omitted from Figure 1. Please add tethering factor CTIF and DDX19B and their correct location relative to the transcript so the reader can follow the progression of mRNA complex formation. This will likely mean moving the newly transcribed mRNAs to the right of the nucleus.

2.	After phosphorylation of UPF1 is introduced in line 85 pg. 4, please remind the reader that phospho-UPF1 binds eIF3 to repress translation initiation.

3.	Sentence 97 on pg.5 is confusing. "Notably, this inhibition is incomplete in non-polarized cells since NMD, and thus translation, occurs." How can a degraded mRNA be translated? Please revise this sentence.

4.	Line 130 page 6 is confusing: what are "non-polarized neurons?"

5.	Line 143 page 7, I think the authors meant to write "bouton" instead of "button."

6.	Lines 252-255, page 11. I do not agree with the sentence describing the data in the bottom of Figure 2 "A similar polarized hierarchy was obtained for mRNAs expressed in hippocampal A1 pyramidal neurons…." The only significant difference in the dendrites is between the NMD-CLIP-FMRP and Others and NMD-CLIP-FMRP and CLIP-FMRP. Thus, it appears that the differences in dendrites are more subtle than those observed in axons, rather than "similar."

7.	Line 263 pg 12, It is hard to believe that Figure 2 supports the idea that direct binding to FMRP and NMD promotes mRNA localization to neuronal projects. It seems that the data show that FMRP/NMD bound mRNAs are more likely to be localized to neuronal projections.

8.	The idea that FXR2 might compensate for FMRP loss is reasonable but it should be noted that FXR2 does not bind G-quadruplexes (Darnell 2009) and does not have an RGG box (Zhang, EMBO J, 1995). Thus, the authors may want to comment on how FXR2 would bind the mRNA cargoes-perhaps through the KH2 domain?

9.	Line 316 page 13, how can degradation by NMD be followed by a burst of localized protein expression? If the mRNA is gone, how can it be translated?

**Reviewer 2**

**Comments to author:**

The manuscript entitled "FMRP-mediated spatial regulation of NMD in neuronal cells", Kurosaki et al summarize previous findings, analyze published datasets, and propose that FMRP binds to and suppress NMD of a fraction of transcripts that are localized in polarized cells. While the study raises an interesting question, the evidence needs to be further refined and rigorously tested/selected/interpreted to enhance the rigor of this work.

1)	The selective association of FMRP and localized NMD transcripts in polarized cells/neurons is interesting. Are there any clues about the mechanism? For example, Is FMRP specifically expressed or modified in polarized cells? Does FMRP selectively bind to specific NMD targets through unique motifs/sequences?

2)	One of the major pieces of evidence to support the authors' claim was the enrichment of NMD transcripts (539/1027) in FMRP targets (5733). Usually, there are about 10,000 genes expressed in any given biological sample, and FMRP appears to bind ~50% of expressed genes. Thus, it is not surprising that ~half of the NMD targets (539/1027) are FMRP targets. However, the authors showed that 539/1027 was a highly significant enrichment with p < 2.2 x 10^-16. How was the enrichment determined here?

3)	Line 250-255: it appears the major factor associated with high axons:somata ratio was FMRP binding. Is there really selectivity for NMD exons here?

Line 265 and the following sections of results: when FMRP was knocked out in the brain or neurons, the FMRP-mediated spatial regulation of NMD transcripts did not show a significant localization change in polarized cells. This is strong evidence against the authors' conclusions. Considering that FMRP has a function in translation inhibition, how to reconcile this observation?

4)	Related to #3 above, there are multiple places where the conclusions are speculative and in certain cases the evidence is even against the authors' claim. I would recommend the authors be more rigorous, accurate, and selective in choosing evidence and drawing conclusions.

For example, line 80: "…providing cells with the ability to better adapt to changes in their environment during development, differentiation and stress, including neurogenesis."

Line188: "…longer 3′UTRs would conceivably allow for more FMRP recruitment/stabilization via more UPF1 and p-UPF1 binding"

Line223-224: stringent thresholds of NMD should have enhanced the true signal instead of limiting

Line225: more NMD exon discovery does not necessarily mean a higher ration of overlap with FMRP targets.

5)	What is the biological significance of the pioneer round of translation of NMD transcripts? The authors speculated (line 120) the burst translation/degradation and their coupling with environmental changes, but in most cases, the NMD transcripts such as the ones caused by alternative splicing, do not produce functional proteins. What are the perspective on this single-round translation of a frequently non-functional protein?

Other points:

6)	1027 NMD targets in N2a cells were identified by Upf1 knockdown and p-Upf1 RIP-sequencing. In addition to NMD regulation, Upf1 has functions in mRNA processing such as releasing mRNA from the gene loci; therefore, mRNAs that are upregulated after Upf1 KD and bound with Upf1 are not necessarily NMD targets. Do they have the characteristics of NMD, such as the distance between the stop codon and EJC, and the sequence/structure of 3'UTR? Or have they been validated by biochemical assays?

7)	Line 424-432: Are there data showing the extent of differentiation? How to define/quantify the differentiation extent? In addition to the differentiation extent, are there other potential causes for the different results in CAD cells and neurons?

**Authors Response**

**Point-by-point responses to the reviewers’ comments:**

We have also addressed the comments from each Reviewer in point-by-point fashion. Our responses begin with “*”.

REVIEWER 1

This important manuscript provides evidence for a molecular model for the bursts of protein translation that are critical for neuronal function. The authors propose that a complex of FMRP and NMD proteins bound to mRNAs modulate a burst of translation at the synapse followed by RNA degradation. As the majority of NMD studies have been conducted in cell lines, it was critically important to examine its role in brain by mining existing data sets from neuronal tissues and substructures. Accordingly, the authors provide evidence for their model by mining a large number of datasets from other labs, as well as their own.

* Our response: We are grateful that this Reviewer appreciates the current need to study physiologic NMD targets in polarized cells from neuronal tissues, which this review argues for and begins to do. Please note that, per the editor’s advice, we have followed the format of a review, beginning with an Introduction subsequent to a 100-word Abstract, and ending with a section entitled “Concluding remarks and future perspectives”.

Although clever and likely true, in part, a problem with this model is that mRNAs must then be continuously supplied by the cell body to the extreme reaches of the neuronal processes. It seems much more efficient to translate an mRNA localized to a distal sight and then stop translation. It would be helpful if the authors addressed this concern. Specifically, the authors should justify why it is important to degrade the mRNA (through NMD), rather than just suppress or cease its translation.

* Our response: Yes, existing data indicate that the model that we put forward is likely true. Please note that, as already shown for the NMD target Arc mRNA (was ref 83; now ref 10; Giorgi et al., Cell 2007), NMD targets, which we have shown are bound by FMRP (ref 3; Kurosaki et al., Nat Cell Biol. 2021) are degraded once translated in response to synaptic activation.

We now reference Rob Singer’s recent paper (now ref 101; Das S, Lituma PJ, Castillo PE, Singer RH. Maintenance of a short-lived protein required for long-term memory involves cycles of transcription and local translation. Neuron. 2023 Jul 5;111(13):2051-2064), which was published while our paper was under review. Das et al. showed for Arc mRNA that a single burst in neuronal stimulation induces cycles of transcriptional reactivation in the same neuron that depend on the previously made ARC protein. The authors concluded that “cycles of transcription-translation coupling sustain protein expression and provide a mechanism by which a short-lived event may support long-term memory.”

We now add the following to what was page 16, line 353 and is now page 18, line 411. “Addressing the issue of how a short-lived burst in neuronal stimulation could support long-term memory, real-time imaging of Arc mRNA movement in individual neurons demonstrated that a single stimulatory event was adequate to induce synapse-localized ARC protein synthesis at so-called “translational hubs”, from which the resulting newly made ARC protein then fed back to induce Arc gene transcription [101]. Notably, some Arc mRNA at these translational hubs appears to be associated with eIF4E at the 5'-cap [102], which likely reflects mRNAs having escaped or already having undergone pioneer rounds of translation either in transit or once localized to the hub.”

Regarding “why it is important to degrade the mRNA (through NMD)”, this is an etiological question that undoubtedly reflects how cells and NMD have co-evolved. However, as we pointed out in our initial submission, once FMRP is released from binding to a projection-localized mRNA, the pioneer round of translation occurs. We are unaware of evidence indicating that once FMRP binding is lost and translation occurs, a subsequent step of translational repression could take place to silence the mRNA. However, the pioneer round of translation followed by NMD requires p-UPF1-mediated translation repression of subsequent rounds of translation, indicating that the repression of translation per se is tightly coupled to the process of NMD. Please note that we now add the following to what is now page 4, lines 82-84. “Phosphorylated UPF1 (p-UPF1) binds to eIF3 of any 48S pre-initiation complex bound to the NMD target’s AUG initiation codon to repress further rounds of translation initiation [33]”.

1. Some proteins described in the text are omitted from Figure 1. Please add tethering factor CTIF and DDX19B and their correct location relative to the transcript so the reader can follow the progression of mRNA complex formation. This will likely mean moving the newly transcribed mRNAs to the right of the nucleus.

* Our response: In Fig. 1a, we now include CTIF and DDX19B on the cytoplasmic side of the nuclear envelop.

2. After phosphorylation of UPF1 is introduced in line 85 pg. 4, please remind the reader that phospho-UPF1 binds eIF3 to repress translation initiation.

* Our response: We previously stated the following on page 3. “Subsequently, a choreographed series of protein−protein rearrangements takes place on the NMD target [13,16,17]. These include recruitment and phosphorylation of the key NMD factor UPF1, which is an ATP-dependent RNA helicase that represses further translation initiation [18] and recruits mRNA degradative activities [19].” As noted above, we have now added the following to the revised manuscript on page 4, lines 82-84. “Phosphorylated UPF1 (p-UPF1) binds to eIF3 of any 48S pre-initiation complex bound to the NMD target’s AUG initiation codon to repress further rounds of translation initiation [33].”.

3. Sentence 97 on pg.5 is confusing. "Notably, this inhibition is incomplete in non-polarized cells since NMD, and thus translation, occurs." How can a degraded mRNA be translated? Please revise this sentence.

* Our response: It is the inhibition of degradation that is incomplete since the inhibition of translation is incomplete. We have now changed the sentence on page 6, lines 122-123 to the following. “Importantly, since NMD, and thus translation, occurs in non-polarized cells, it follows that the FMRP-mediated inhibition of translation is incomplete in these cells.”.

4. Line 130 page 6 is confusing: what are "non-polarized neurons?"

* Our response: This is our oversight, for which we apologize. We have now changed “non-polarized neurons” to “non-polarized cells”.

5. Line 143 page 7, I think the authors meant to write "bouton" instead of "button."

* Our response: We have changed “button-like terminals” to “button-like terminals called “boutons””.

6. Lines 252-255, page 11. I do not agree with the sentence describing the data in the bottom of Figure 2 "A similar polarized hierarchy was obtained for mRNAs expressed in hippocampal A1 pyramidal neurons…." The only significant difference in the dendrites is between the NMD-CLIP-FMRP and Others and NMD-CLIP-FMRP and CLIP-FMRP. Thus, it appears that the differences in dendrites are more subtle than those observed in axons, rather than "similar."

* Our response: We have now changed to the following on pages 12-13, lines 282-285. “A more subtle and slightly different polarized hierarchy was obtained for mRNAs expressed in hippocampal CA1 pyramidal neurons [12], i.e. enrichment of “NMD + CLIP-FMRP targets” > “NMD targets” > “CLIP-FMRP targets” > “Others”, in dendrites relative to somata (Fig. 2d,e).”

7. Line 263 pg 12, It is hard to believe that Figure 2 supports the idea that direct binding to FMRP and NMD promotes mRNA localization to neuronal projects. It seems that the data show that FMRP/NMD bound mRNAs are more likely to be localized to neuronal projections.

* Our response: We believe that this Reviewer is referring to the sentences: “Our findings refine their conclusion by showing that FMRP-bound NMD targets are preferentially enriched in axons and dendrites relative to somata when compared to FMRP targets that are not high-confidence NMD targets or NMD targets that do not CLIP with FMRP. In other words, our results suggest that both direct binding of FMRP to cellular mRNAs and binding of FMRP via UPF1 and p-UPF1 to cellular NMD targets promote mRNA localization to neuronal projections in a cumulative manner.”

Note that it is not NMD that promotes localization, but how the mRNAs are recognized as NMD targets, which we propose is by the UPF1- and p-UPF1-mediated recruitment of FMRP prior to translation and NMD. Taking into consideration this Reviewer’s comment #6, we added the following to page 13, lines 293-298. “Nonetheless, relative to UPF1- and p-UPF1-mediated binding, direct binding by FMRP to target mRNAs is a bigger contributor to the enrichment of mRNAs in axons. Conversely, while statistical analyses are less conclusive, it appears that relative to direct binding, UPF1- and p-UPF1-mediated binding by FMRP to target mRNAs is a bigger contributor to the enrichment of mRNAs in dendrites.”

8. The idea that FXR2 might compensate for FMRP loss is reasonable but it should be noted that FXR2 does not bind G-quadruplexes (Darnell 2009) and does not have an RGG box (Zhang, EMBO J, 1995). Thus, the authors may want to comment on how FXR2 would bind the mRNA cargoes-perhaps through the KH2 domain?

* Our response: We have now done this. We had stated the following on what was page 13, line 289 and is now pages 14, line 325. “In further support of this possibility, RNA-binding properties of FMRP and FXR2 KH2 domains are very similar [86]. Moreover, for NMD targets, FXR2, like FMRP, co-immunoprecipitates with p-UPF1 in an RNase I-resistant manner using HEK293T-cell lysates [3]. However, since FXR2 lacks an RGG domain [80], if and how FXR2 could be recruited to non-NMD targets that FMRP binds via G-quadruplexes remains unclear.”

9. Line 316 page 13, how can degradation by NMD be followed by a burst of localized protein expression? If the mRNA is gone, how can it be translated?

* Our response: The burst of localized protein expression is the result of rapid translational derepression, allowing for the pioneer round of translation followed by rapid NMD. We rephrased our writing as follows on page 16, lines 370-374: “Thus, we hypothesize that for NMD targets, eventual release from FMRP-mediated translational repression in axons is accompanied by protein production during the pioneer round of translation and, as a consequence, rapid mRNA degradation by NMD. This ensures a burst of localized protein expression.”

We thank this Reviewer for helping us to clarify important points in our review.

REVIEWER 2

The manuscript entitled "FMRP-mediated spatial regulation of NMD in neuronal cells", Kurosaki et al summarize previous findings, analyze published datasets, and propose that FMRP binds to and suppress NMD of a fraction of transcripts that are localized in polarized cells. While the study raises an interesting question, the evidence needs to be further refined and rigorously tested/selected/interpreted to enhance the rigor of this work.

* Our response: Please note that this is not a research article, but a review of existing literature that we enrich with new analyses of available datasets. While we are currently not in the position to work with polarized cells at the level that this Reviewer suggests we do, we hope that, by supplementing our review with literature that has been published since our initial submission to Genome Biology, and by clarifying our methodology, our review is even more compelling. Additionally, per the editor’s advice, we have followed the format of a review, beginning with an Introduction subsequent to a 100-word Abstract, and ending with a section entitled “Concluding remarks and future perspectives”.

1) The selective association of FMRP and localized NMD transcripts in polarized cells/neurons is interesting. Are there any clues about the mechanism? For example, Is FMRP specifically expressed or modified in polarized cells? Does FMRP selectively bind to specific NMD targets through unique motifs/sequences?

* Our response: FMRP binds to virtually all NMD targets either directly, preferring GC-rich and/or structured sequences that are also preferred by UPF1 and p-UPF1, or via UPF1 and p-UPF1, the latter of which may involve FMRP binding indirectly to NMD targets. While we discussed the UPF1- and p-UPF1-mediated binding of FMRP possibly being indirect binding to RNA, we did not describe the direct binding-site preference of FMRP, which we now do on pages 5-6, lines 119-122 as follows. “FMRP can also bind directly to NMD targets, as it does to non-NMD targets, at G-rich and/or structured sequences, which are sequences that also typify UPF1- and p-UPF1-binding sites in NMD targets [4 and references therein].”

We later discuss how FMRP and NMD targets are selectively localized and, upon the phosphorylation of FMRP in response to synaptic activity, translated and then degraded by NMD in projections. This is the point of mining data that used polarized cells and derive from others. Please see sections entitled, e.g. “FMRP-bound NMD targets most efficiently localize to neuronal projections”, and “FMRP represses most efficiently the translation of FMRP-bound NMD targets”.

2) One of the major pieces of evidence to support the authors' claim was the enrichment of NMD transcripts (539/1027) in FMRP targets (5733). Usually, there are about 10,000 genes expressed in any given biological sample, and FMRP appears to bind ~50% of expressed genes. Thus, it is not surprising that ~half of the NMD targets (539/1027) are FMRP targets. However, the authors showed that 539/1027 was a highly significant enrichment with p < 2.2 x 10^-16. How was the enrichment determined here?

* Our response: Please note that we have previously shown, using RIP-seq (which does not require that FMRP bind directly to mRNA) that essentially all NMD targets are associated with FMRP (Kurosaki et al., Nat Cell Biol. 2021). We had stated this in several places. We also wrote on what is now page 10, lines 233-235, the following. “Notably, among these NMD targets, all tested were found to be destabilized in Fmr1-KO P1 cortical neurons [32], reinforcing the idea that FMRP generally stabilizes NMD targets, which it binds via UPF1 or p-UPF1 [3].”.

We go on to explain on page 11, lines 248-250 “why only 539 NMD targets identified for mouse N2A cells overlap with the 5,733 CLIP-FMRP targets that were identified using six different mouse brain-derived transcriptomes.”. Yes, it is not surprising based on our RIP-seq data that 539/1027 was a highly significant enrichment, but we needed to do likewise for CLIP-seq data given the limitation that CLIP-seq measures only mRNAs that are directly bound by FMRP.

In response to how enrichment was determined, we used transcripts from the 21,565 protein-coding genes from the mouse GENCODE M32 database (https://www.gencodegenes.org/mouse/stats_M32.html) as our starting pool. This is because the transcriptome for each mouse cell line and tissue that we analyzed has not been completely characterized and is expected to vary when comparing the cell lines and tissues. We found that the odds of “NMD targets” being among “CLIP-FMRP targets” are about three-fold above the odds of “NMD targets” being among non-CLIP-FMRP targets. We now state the following in the Fig. 2a legend. “The statistical significance of the overlap between “NMD targets” and “CLIP-FMRP targets”, i.e. “NMD + CLIP-FMRP targets”, in the pool of 21,565 protein-coding genes derived from the mouse GENCODE M32 database (https://www.gencodegenes.org/) was calculated using Fisher’s exact test (P < 2.2 x 10-16, odds ratio = 3.26, 95% confidence interval 2.87-3.71) in R version 3.6.3 [116].”.

3) Line 250-255: it appears the major factor associated with high axons:somata ratio was FMRP binding. Is there really selectivity for NMD exons here?

* Our response: We see that both direct FMRP binding (“CLIP-FMRP targets”) and what may be indirect FMRP binding, i.e. through UPF1 and p-UPF1 (“NMD targets) contribute to localize mRNAs to axons and dendrites. Please see our response to Reviewer 1’s comments #6 and #7 for the relative contributions of direct and UPF1- and p-UPF1-mediated FMRP binding to transcript enrichment in axons and dendrites. As stated throughout the manuscript, and as we have published (Kurosaki et al., Nat Cell Biol. 2021; Kurosaki et al., Moll Cell 2022), FMRP binding largely stabilizes both NMD and non-NMD targets. We show here that NMD targets constitute the category of mRNAs that are most efficiency localized to and stabilized at neuronal projections by FMRP, which is the focus of this Review.

Line 265 and the following sections of results: when FMRP was knocked out in the brain or neurons, the FMRP-mediated spatial regulation of NMD transcripts did not show a significant localization change in polarized cells. This is strong evidence against the authors' conclusions. Considering that FMRP has a function in translation inhibition, how to reconcile this observation?

* Our response: As noted in what were lines 256-258 and 267-270, and are now lines 286-288 and 302-305 on page 13, our conclusions agree with conclusions made by other laboratories. In an attempt to reconcile apparently incompatible results, we propose partial functional redundancy between FMRP and other RNA-binding proteins, for example FXR2. Please note that Reviewer 1 mentions that “The idea that FXR2 might compensate for FMRP loss is reasonable”. We also moved from the Discussion to page 15 a paragraph about the possibility of FMRP functional redundancy with TDP-43.

FMRP may promote enrichment of NMD and non-NMD targets to projections not only by promoting the transport of these mRNAs to projections but also by repressing their translation (and thus their decay) in projections, where FMRP is dephosphorylated in response to stimuli. While FXR2 was reported to repress the translation of at least some mRNAs, it is unclear if it represses all mRNAs that it binds. Additionally, it is likely that the relative activities of FXR2 and FMRP are different in somata relative to in projections. Thus, functional compensation of FMRP loss by FXR2 and/or other proteins is complex, and addressing it further is beyond the scope of this review.

4) Related to #3 above, there are multiple places where the conclusions are speculative and in certain cases the evidence is even against the authors' claim. I would recommend the authors be more rigorous, accurate, and selective in choosing evidence and drawing conclusions.

* Our response: We agree that some conclusions are speculative, and we state so in the manuscript. As examples, we use the phrases “our results suggest”, “these findings suggest”, “in support of this possibility”, “if and how FXR2 could be”, “a case could be made”, and so on.

For example, line 80: "…providing cells with the ability to better adapt to changes in their environment during development, differentiation and stress, including neurogenesis."

* Our response: We do not understand how this Reviewer can conclude that evidence exists against this claim. Please see references 8 and 7, which are reviews from our lab or from the labs of Jaffrey and Wilkinson, each of which offers examples of NMD-mediated adaptation via changes in NMD efficiency, including during neurogenesis (the latter of which we also showed during mouse brain development in utero and post-birth in Kurosaki et al., Genome Biology, 2021).

Line188: "…longer 3′UTRs would conceivably allow for more FMRP recruitment/stabilization via more UPF1 and p-UPF1 binding"

* Our response: Line 188 is now line 214. Indeed, “long” (we refer to them as long and/or structured) 3'UTRs trigger NMD. This reasoning is based on current knowledge in the field (as examples, Eberle et al., PLoS Biol. 2008; Singh et al., PLoS Biol. 2008; Imamachi et al., Genome Res. 2017; Kurosaki et al., Gene Dev. 2014; and Kurosaki et al., Nat Struct Mol Biol. 2018). On page 4, lines 95-96, we reference all of these papers indirectly in references 8 and/or 42, which are two reviews: a 2016 review from Oliver Mühlemann’s lab and a 2019 review from our lab.

Please note that we are unsure how this finding is counter to our claims. All of the studies just referenced were performed using non-polarized cells in which FMRP binding to an NMD target – even NMD targets with a long and/or structured 3'UTR – does not completely inhibit NMD (Kurosaki et al., 2021, Nat. Cell Biol.; Kurosaki et al., 2022, Mol. Cell). We stated this on what are now page 6, lines 122-123. Thus, since one hallmark of cellular mRNAs that are physiologic NMD targets is a long and/or structured 3'UTR, the degree to which FMRP is recruited to longer 3'UTRs by UPF1 and p-UPF1, which this Reviewer is correct in thinking would exceed the degree of FMRP recruitment to shorter 3'UTRs, is still insufficient to nullify the NMD mechanism through translational repression. In polarized, one would expect that the FMRP-mediated inhibition of the pioneer round of translation would occur until an NMD target with a longer 3'UTR is localized to projections and translationally activated by FMRP loss, at which point NMD would occur.

Line 223-224: stringent thresholds of NMD should have enhanced the true signal instead of limiting

* Our response: It is unclear why this Reviewer states that working with a stringent set of NMD targets limits the signal. We simply claimed that the list of “NMD targets” was likely underestimated, as detailed on what used to be page 10, beginning on line 222, and is now page 11, beginning on line 252.

Line225: more NMD exon discovery does not necessarily mean a higher ration of overlap with FMRP targets.

* Our response: While defining additional NMD targets would not necessarily increase the fraction of NMD targets that are directly bound by FMRP, it would most likely increase the fraction of FMRP-bound mRNAs that are NMD targets, i.e. the limitation cited in what used to be page 10, lines 217-220, and is now page 11, lines 247-250.

5) What is the biological significance of the pioneer round of translation of NMD transcripts? The authors speculated (line 120) the burst translation/degradation and their coupling with environmental changes, but in most cases, the NMD transcripts such as the ones caused by alternative splicing, do not produce functional proteins. What are the perspective on this single-round translation of a frequently non-functional protein?

* Our response: The biological significance of the pioneer round of translation for NMD targets, as addressed in our manuscript, pertains to natural, physiologic mammalian-cell mRNAs. Please note that our focus is not on the NMD-mediated degradation of mRNAs encoding non-functional proteins.

We had written the following on lines 79-83. “NMD also degrades 5-10% of natural, physiologic mammalian-cell mRNAs, providing cells with the ability to better adapt to changes in their environment during development, differentiation and stress, including neurogenesis [26, 30]. These targets can harbor an upstream open translational reading frame (uORF) or result from regulated alternative pre-mRNA splicing and/or 3'-end formation [31, 32].” To clarify further, we have modified these sentences, now on lines 105-110, as follows. “NMD also degrades 5-10% of natural, physiologic mammalian-cell mRNAs, providing cells with the ability to better adapt to changes in their environment during stress, development, and differentiation, including neurogenesis [7,8]. This class of physiologic NMD targets, which is the focus of this review, can harbor an upstream open translational reading frame (uORF) or result from regulated, i.e. constructive, alternative pre-mRNA splicing and/or 3'-end formation [1,8,46].”.

So that others are not confused, we additionally change the title of our Review from “FMRP-mediated spatial regulation of NMD in neuronal cells” to “FMRP-mediated spatial regulation of physiologic NMD targets in neuronal cells”. And, we changed “natural” NMD targets to “physiologic” NMD targets in several places. The abstract has also been changed accordingly, and shortened to the 100-word limit for a Review.

Other points:

6) 1027 NMD targets in N2a cells were identified by Upf1 knockdown and p-Upf1 RIP-sequencing. In addition to NMD regulation, Upf1 has functions in mRNA processing such as releasing mRNA from the gene loci; therefore, mRNAs that are upregulated after Upf1 KD and bound with Upf1 are not necessarily NMD targets. Do they have the characteristics of NMD, such as the distance between the stop codon and EJC, and the sequence/structure of 3'UTR? Or have they been validated by biochemical assays?

* Our response: We are aware of the paper describing that UPF1 functions to release mRNA from the gene loci. This work pertains to S. pombe and derives from the lab of Saverio Brogna (De S, Edwards DM, Dwivedi V, Wang J, Varsally W, Dixon HL, Singh AK, Owuamalam PO, Wright MT, Summers RP, Hossain MN, Price EM, Wojewodzic MW, Falciani F, Hodges NJ, Saponaro M, Tanaka K, Azzalin CM, Baumann P, Hebenstreit D, Brogna S. Genome-wide chromosomal association of Upf1 is linked to Pol II transcription in Schizosaccharomyces pombe. Nucleic Acids Res. 2022 Jan 11;50(1):350-367). We have no evidence that UPF1 or p-UPF1 functions in mammalian cells in the nucleus to directly regulate mRNA processing. Furthermore, even if this mechanism were conserved in mammals, UPF1 knockdown would result in the downregulation of nuclear precursors to NMD targets, which we do not see, while upregulating NMD targets.

Please note that Brogna also published that in Drosophila there is translation in the nucleus, that EJCs can be deposited independently of splicing, and that there is a “slight” RNA polymerase II pausing upon UPF1 depletion in D. melanogaster (Singh AK, Zhang J, Hebenstreit D, Brogna S. Evidence of slightly increased Pol II pausing in UPF1-depleted Drosophila melanogaster cells. MicroPubl Biol. 2020 Oct 16;2020:10.17912/micropub.biology.000319). There are lots of data deriving from mammalian cells that counter these claims, including most recently unpublished data from Hanae Sato (a former post-doc in my lab) with Rob Singer that UPF1 knockdown does not cause detectable RNA polymerase II pausing (personal communication).

7) Line 424-432: Are there data showing the extent of differentiation? How to define/quantify the differentiation extent? In addition to the differentiation extent, are there other potential causes for the different results in CAD cells and neurons?

* Our response: We agree with the inference of these questions: that it is difficult to differentiate cells in vitro to the extent that cells do in an animal. The CAD mouse neuronal-cell data (Fig. S2) derive from Goering et al. (previous ref 96; new ref 113). While comparative analyses of the differentiated CAD cells and neurons were not performed, we now add the following to the Fig. S2 legend. “CAD cells were induced to differentiate into a more neuronal state through the withdrawal of serum for 48 hr.”

We also thank this Reviewer for helping us to clarify important points in our review.

**Second round of review**

**Reviewer 1**

This significantly improved manuscript was a delight to read. It was both novel and informative and did an excellent job of identifying conflicting studies with possible explanations for the differences.

My only suggestion would be to move Supplementary Figures 1a and b into Main Figure 3 because they demonstrate the decreased mRNA abundance of the translated NMD targets, supporting the model that after translation, the mRNAs are degraded.
